# The lack of evidence behind over-the-counter antioxidant supplements for male fertility patients: a scoping review

**DOI:** 10.1093/hropen/hoad020

**Published:** 2023-05-17

**Authors:** Wiep R de Ligny, Kathrin Fleischer, Hilde Grens, Didi D M Braat, Jan Peter de Bruin

**Affiliations:** Department of Reproductive Medicine, Radboud University Medical Center, Nijmegen, The Netherlands; Nij Geertgen Center for Reproductive Medicine, Elsendorp, The Netherlands; Center for Reproductive Medicine, Jeroen Bosch Hospital, ‘s-Hertogenbosch, The Netherlands; Department of Reproductive Medicine, Radboud University Medical Center, Nijmegen, The Netherlands; Center for Reproductive Medicine, Jeroen Bosch Hospital, ‘s-Hertogenbosch, The Netherlands

**Keywords:** male infertility, antioxidants, supplements, oxidative stress, vitamins

## Abstract

**STUDY QUESTION:**

What is the evidence for over-the-counter antioxidant supplements for male infertility?

**SUMMARY ANSWER:**

Less than half of over-the-counter antioxidant supplements for male fertility patients have been tested in a clinical trial, and the available clinical trials are generally of poor quality.

**WHAT IS KNOWN ALREADY:**

The prevalence of male infertility is rising and, with this, the market for supplements claiming to improve male fertility is expanding. Up to now, there is limited data on the evidence for these over-the-counter supplements.

**STUDY DESIGN, SIZE, DURATION:**

Amazon, Google Shopping and other relevant shopping websites were searched on 24 June 2022 with the following terms: ‘supplements’, ‘antioxidants’, ‘vitamins’, AND ‘male fertility’, ‘male infertility’, ‘male subfertility’, ‘fertility men’, ‘fertility man’. All supplements with a description of ingredients in English, Dutch, French, Spanish, or German were included. Subsequently, Pubmed and Google Scholar were searched for studies that included the supplements.

**PARTICIPANTS/MATERIALS, SETTING, METHODS:**

Inclusion criteria were supplements with antioxidant properties, of which the main purpose was to improve male fertility. Included supplements must be available without a doctor’s prescription. Supplements containing plant extracts were excluded, as well as supplements of which the content or dosage was not clear. The ingredients, dosage, price and health claims of the supplements were recorded. We assessed whether substances in the supplements exceeded the recommended dietary allowance (RDA) or tolerable upper intake level (UL). All clinical trials and animal studies investigating included supplements were selected for this review. Clinical trials were assessed for risk of bias with a risk of bias tool appropriate for the study design.

**MAIN RESULTS AND THE ROLE OF CHANCE:**

There were 34 eligible antioxidant supplements found, containing 48 different active substances. The average price per 30 days was 53.10 US dollars. Most of the supplements (27/34, 79%) contained substances in a dosage exceeding the recommended daily allowance (RDA). All manufacturers of the supplements made health claims related to the improvement of sperm quality or male fertility. For 13 of the 34 supplements (38%), published clinical trials were available, and for one supplement, only an animal study was found. The overall quality of the included studies was poor. Only two supplements were tested in a good quality clinical trial.

**LIMITATIONS, REASONS FOR CAUTION:**

As a consequence of searching shopping websites, a comprehensive search strategy could not be formulated. Most supplements were excluded because they contained plant extracts or because supplement information was not available (in an appropriate language).

**WIDER IMPLICATIONS OF THE FINDINGS:**

This is the first review that gives an insight into the market of male fertility supplements as available to infertility patients and other men seeking to improve their fertility. Earlier reviews have focused only on supplements with published clinical trials. However, we show that more than half of the supplements have not been tested in a clinical trial. To our knowledge, this review is the first to assess the dosage of supplements in relation to the RDA. In agreement with the literature, we found that the evidence on male fertility supplements is generally of poor quality. This review should urge pharmaceutical companies to evaluate their products in randomized controlled trials in order to provide people with substantiated information.

**STUDY FUNDING/COMPETING INTEREST(S):**

The research position of W.R.d.L. is funded by an unrestricted grant from Goodlife Pharma. W.R.d.L., K.F., and J.P.d.B. are in the research team of a clinical trial on Impryl^®^, one of the supplements included in this review.

**REGISTRATION NUMBER:**

N/A.

WHAT DOES THIS MEAN FOR PATIENTS? This review looks at which antioxidant (nutritional) supplements are freely available to men who want to improve their fertility. We assessed whether they are safe and whether research has proved their effect.

In this study, we searched shopping websites for supplements that claim to improve the fertility of men. We found a total of 34 different supplements. Most (79%) of the supplements contained excessive dosages of vitamins or minerals, which may cause lower semen quality. We also found that a minority (38%) of the supplements had been tested in a published study. If a supplement was tested in a study, the study was generally of poor quality.

## Introduction

Infertility is a widespread problem affecting 8–12% of the population worldwide (Vander Borght and Wyns, 2018). In around 50% of these couples, a male factor is involved, and in 20–30%, it is the main cause of infertility (Agarwal *et al.*, 2021). Some studies have reported a decline in semen quality over the past decades (Sengupta *et al.*, 2017; Agarwal *et al.*, 2021; Levine *et al.*, 2023). Researchers have hypothesized chemical substances (e.g. pesticides), radiation and heat, increases in BMI, advanced male age, and consumption of alcohol and tobacco, as possible causes (Sengupta *et al.*, 2017; Darbandi *et al.*, 2018). However, the meta-analyses reporting a decline in semen quality have been criticized for their methodological limitations (Bonde and Te Velde, 2017). Other studies have not found evidence for this decline or have shown large geographical variations (Swan *et al.*, 1997, 2000; Cipriani *et al.*, 2023). Either way, the recognition of the importance of male (in)fertility has increased and in reaction to this, various (pharmaceutical) companies have developed over-the-counter (OTC) nutritional supplements.

The use of supplements, antioxidant supplements in particular, to improve male fertility is based on the process of oxidative stress (Agarwal *et al.*, 2019). Oxidation occurs in all cells in the human body. In oxidation, a substance loses electrons, which can result in the production of free radicals or reactive oxygen species (ROS). ROS, in turn, can damage (parts of) the sperm cell. Reduction is the process of substances gaining electrons, the opposite of oxidation. The reaction between an oxidative agent and a reductive agent is called a redox reaction.

Antioxidants can prevent the production of ROS or inactivate ROS before they can damage the cell, its DNA or other cell components. When there is an imbalance between oxidation (i.e. the production of ROS) and the level of antioxidants, oxidative stress occurs. Sperm cells are particularly vulnerable to oxidative stress. This is firstly because the sperm cell membrane is rich in polyunsaturated fatty acids, which oxidize easily. Secondly, sperm cells contain little antioxidant-rich cytoplasm.

Building on this theory, antioxidant supplements could prevent or treat oxidative stress in sperm cells and thus improve male fertility. The updated Cochrane review on antioxidants for male subfertility showed that live birth rate slightly increased when antioxidants were used. However, this effect could not be shown when studies with a high risk of bias were removed from the analysis (de Ligny *et al.*, 2022).

It is important to note that a crucial role of oxidative stress is still a matter of debate among male fertility experts. Moreover, it has been reported that the excessive use of antioxidants can result in impaired fertility through the process of reductive stress (Dattilo *et al.*, 2016; Henkel *et al.*, 2019). Antioxidants should therefore be handled with caution in the treatment of male infertility.

As mentioned above, many companies have developed OTC antioxidant supplements that claim to improve male fertility. Some companies have even been founded to focus only on supplements for improving female and/or male fertility. These companies meet the need of fertility patients searching for alternative or additional treatment options. However, it is often unknown whether there is scientific evidence for these specific supplements. If evidence is lacking, this could lead to overtreatment or even impairment of male fertility when reductive stress occurs (Dattilo *et al.*, 2016; Henkel *et al.*, 2019). Moreover, it can lead to serious costs for patients.

Ideally, all supplements that are available to patients and that claim to improve fertility should have been tested in large randomized controlled trials (RCTs). It is of great importance that these trials have relevant outcomes such as clinical pregnancy rates and live birth rates, as these are the ultimate goals for fertility patients and their health care providers. This is also reflected in the infertility core outcome set as developed by Duffy *et al.* (2021).

The aim of this scoping review is to assess the available OTC antioxidant supplements for male subfertility, and to evaluate the scientific basis of health claims.

## Materials and methods

### Search

On 24 June 2022, the websites of Google Shopping (‘Google Shopping’) and Amazon (‘Amazon’) were searched with the following terms: ‘supplements’, ‘antioxidants’, ‘vitamins’ AND ‘male fertility’, ‘male infertility’, ‘male subfertility’, ‘fertility men’, ‘fertility man’. While conducting this search, different websites for supplements were identified and subsequently searched for new supplements. The websites of manufacturers of included supplements were searched for other eligible products. Reference lists of reviews and studies of included supplements were explored as well.

### Inclusion and exclusion criteria

Supplements were included based on the following criteria. (i) Supplements must consist of substances with antioxidant properties as defined in the Cochrane review updated in 2022 (de Ligny *et al.*, 2022): ‘Antioxidants are substances that inhibit or delay the oxidation of biologically relevant molecules, either by directly scavenging free radicals or by chelation of redox metals’. (ii) The main purpose of the supplement, according to the manufacturer, must be to improve male fertility. More specifically, this means that the package or container should mention at least one of the following terms: fertility, fertile, infertility, subfertility, pre-conception or reproduction, and male, men, man, him. (iii) The supplement is available without a doctor’s prescription.

Exclusion criteria were as follows. (i) Supplements containing plant extracts were excluded because the dosage as well as the antioxidant properties of plant extracts are often unclear. (ii) Supplements of which the content or dosage were not clear or described in a language other than English, Dutch, French, Spanish, or German, were also excluded. Although the English websites of Google Shopping and Amazon were searched, some supplements contained information in another language.

### Data collection

After identifying all eligible supplements, predefined data on the product were retrieved. Information on the product was obtained from the website of the manufacturer or, if not available, from the website where the product can be purchased. Predefined outcomes were ingredients and the dosage of the supplement, claims regarding male fertility as stated by the manufacturer (written on the website or on the packaging of the product), and price per 30 days in US dollars.

If available, the dosage of individual ingredients was related to the recommended dietary allowance (RDA) and tolerable upper intake level (UL). All ingredients of which an RDA or UL was available, were assessed. Both values were retrieved from the database of the NIH Office of Dietary Supplements (Medicine 2000, 2001, 2011, 2005, 1998). From this database, the highest value for men aged between 18 and 50 years was used.

### Search and risk of bias assessment of studies

Scientific evidence on the supplements was searched on the supplement websites, and on Pubmed and Google Scholar, searching for the (brand) name of the supplement. If this retrieved no articles, the search was repeated with the name of the manufacturer. Only studies investigating a supplement with the exact same composition as the included supplement were evaluated in this review. Studies that did not investigate the supplement in relation to male fertility were not included in this review. Studies in a language other than English, Dutch, French, Spanish, or German, were excluded from this review.

Included studies were then assessed for risk of bias with the appropriate risk of bias tool by two of four independent researchers: W.R.d.L. and J.P.d.B., H.G., or L.M.P. Huygen (who provided assistance with the Spanish studies; see Acknowledgements). For each study design, a distinctive tool was used because different study methods require different methodological quality assessment. For RCTs, non-randomized studies of interventions, and pre–post studies with a control group, the Cochrane Risk of Bias 2 (RoB2), the ROBINS-I and the EPOC RoB tool were used, respectively. For pre–post studies without a control group, the NIH tool was used. For cohort studies, the Newcastle-Ottawa Scale (NOS) was used. These tools were assigned to each study design according to Ma *et al.* (2020). Disagreements were discussed among researchers and if consensus could not be achieved, a third researcher was involved: W.R.d.L., J.P.d.B., H.G., or L.M.P. Huygen (see Acknowledgements).

## Results

### Search and selection of supplements

**Figure 1.**
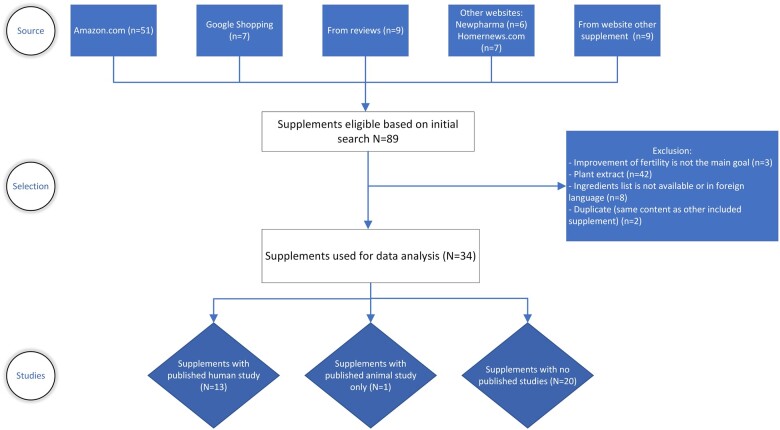
Flow diagram of search and selection of eligible supplements and corresponding studies.

The search yielded a total of 89 eligible supplements based on the product name (Fig. 1). Most of these products (51 supplements) were found on Amazon. Seven supplements were retrieved from the Google Shopping website. Two websites mentioning several supplements for male fertility were also found: Newpharma.nl and Homer News, providing six and seven previously unidentified supplements, respectively. Nine other supplements were found through the websites of included supplements. Reference checking of reviews yielded nine additional supplements.

All 89 supplements were then screened more thoroughly by assessing the package (online) and, if available, the manufacturer’s website. This led to the exclusion of 42 supplements for containing plant extracts (e.g. maca root, pine bark, ginseng, green tea, etc.). Eight supplements were excluded due to unknown content or formulation of ingredients. This was either not mentioned on the packaging or website, or the website was only available in Russian. Two products had exactly the same content and manufacturer as another included supplement and were therefore removed as duplicates. Finally, three supplements were excluded for not having improvement of male fertility as the main goal. Thus, 34 supplements were included for qualitative analysis.

### Characteristics of included supplements

The 34 included supplements contained 48 different active substances. Most of the included supplements (27/34, 79%) contained substances in a dosage exceeding the RDA. Two supplements, Belimen^®^ and FH Pro for men^®^, contained dosages exceeding the tolerable UL (Table 1). The price per 30 days ranged from 21.76 to 159.64 US dollars, with an average of 53.10 US dollars.

**Table 1.** Characteristics of included supplements.

Supplement name	Manufacturer	Ingredients (daily dose)	Dose	Excee-ding RDA	Excee-ding UL	Price/30 days	Claims^a^	Studies (human) Y/N	Studies (animal) Y/N	Claims supported by evidence Y/N^b^	Study references
Androferti^®^	Qpharma	l-Carnitine 1500 mg, Coenzyme Q10 20 mg, Vit C 60 mg, Vit E 10 mg, Zinc 10 mg, FA 200 µg, Selenium 50 µg, Vit B12 1 µg	2/day	N	N	$93.84	It is based on its unique formulation of l-carnitine with antioxidants, vitamins and minerals. ANDROFERTI^®^ components contribute to the normalization of fertility and reproduction, also it has antioxidant action.	Y	N	N	Abad *et al.* (2013), Nazari *et al.* (2021), Balmori Boticario *et al.* (2010), Ballescá *et al.* (2012), López Granollers *et al.* (2011), and Junca *et al.* (2012)
Condensyl^®^	Nurilia	l-Cysteine 170 mg, Betalain 0,05 mg, Quercetine 0.001 mg, Zinc 12,5 mg,^d^ Vit B3 16 mg, Vit E 12 mg, Vit B6 1,4 mg,^d^ Vit B2 1,4 mg,^d^ FA 400 µg, Vit B12 2,5 µg^d^	1/day	Y	N	$28.00	Reducing sperm DNA disorganization involves restoring antioxidant defense and optimizing male fertility.	Y	Y	NA (claim not directly related to the product)	Amar *et al.* (2015), Dattilo *et al.* (2014) (human) Mohammadi *et al.* (2018) (animal)
Cyclofert Man^®^	Laboratoires Surveal	l-Carnitine 250 mg, N-acetylcysteine 150 mg, Vit E 16,5 mg,^d^ Vit C 50 mg, Selenium 50 μg, FA 100 μg, Lycopene 5 mg, Coenzyme Q10 25 mg, Zinc 10 mg	1/day	Y	N	$35.15	Cyclofert Man contains selenium that ensures normal spermatogenesis and zinc that enables normal fertility and reproduction.	Y	N	NA (claim not directly related to the product)	Elmahaishi *et al.* (2017)
Fertilaid for men^®^	Fairhaven health	Vit A 1500 µg,^d^ Vit C 250 µg,^d^ Vit D 10 µg, Vit E 100.5 µg, Vit K 80 µg, Vit B1 1.5 mg,^d^ Vit B2 1.7 mg,^d^ Vit B3 20 mg,^d^ Vit B6 2 mg,^d^ FA 850 µg,^d^ Vit B12 25 µg,^d^ Vit B5 10 mg,^d^ Iodine 150 µg, Magnesium 120 mg, Zinc 30 mg,^d^ Copper 2 mg,^d^ Manganese 2 mg, Chromium 120 µg^d^	3/day	Y	N	$32.95	Popular male fertility supplement since 2003 to support healthy sperm count, motility, and morphology. A 2-in-1 powerful male fertility pill PLUS male prenatal vitamin. Offers a wide spectrum of key fertility ingredients, including l-carnitine, zinc, Coenzyme Q10, and Patented formula to support male reproductive health and fertility.	Y	N	Y (partly: improvement of total motile sperm count)	Clifton and Ellington (2009)
Fertilovit Mplus^®^	Fertilovit	l-Citruline 300 mg, Glutathione 50 mg, N-acetylcysteine 50 mg, Vit C 100 mg,^d^ Vit E 100 mg,^d^ FA 500 µg,^d^ Zinc 25 mg,^d^ Selenium 100 µg,^d^ Sodium 0.03 mg, l-carnitine 300 mg, Coenzyme Q10 15 mg, Lycopene 4 mg	2/day	Y	N	$49.00	The only product worldwide for this application with antioxidants with long-term effects. Tested effective.	Y	N	Y (partly: improvement of total motile sperm count, follow-up 12 months)	Wirleitner *et al.* (2012)
Fertimax2^®^	DCMG	l-Carnitine 400 mg, Vit C 180 mg,^d^ Vit E 30 mg,^d^ Zinc 15 mg,^d^ Selenium 50 µg, FA 200 µg, Coenzyme Q10 40 mg	1/day (one green and one white tablet)	Y	N	$27.95	FERTIMAX 2™ is a dietary supplement specifically formulated to meet the nutritional needs of men wishing to have a child. Thanks to an optimized combination of antioxidants and micronutrients, FERTIMAX ™ 2 contributes to: healthy fertility and reproduction (zinc), normal spermatogenesis (selenium) and synthesis of DNA (zinc), the protection of cells against oxidative stress (vitamins C, E, selenium)	Y	N	N	Kacem *et al.* (2014)
FH PRO for men^®^	Fairhaven health	Vit A 1500 µg,^d^ Vit C 120 mg,^d^ Vit D 30 µg,^d^ Vit E 134 mg,^d^ Vit K 80 µg, Vit B1 3 mg,^d^ Vit B2 3.4 mg,^d^ Vit B3 20 mg,^d^ Vit B6 25 mg,^d^ FA 1360 µg,^e^ Vit B12 1000 µg,^d^ Biotine 600 µg,^d^ Vit B5 20 mg,^d^ Iodine 150 ug, Zinc 30 mg,^d^ Selenium 140 µg,^d^ Copper 1 mg,^d^ Manganese 2 mg, Chromium 120 µg,^d^ Molybdenum 75 µg, l-carnitine 2 g, l-arginine 350 mg, Coenzyme Q10 200 mg, N-acetyl-cysteine 200 mg, Lycopene 10 mg, Benfotiamine 1 mg	6/day	Y	Y	$94.95	Shown in a clinical study to improve sperm count, motility, and morphology … designed to support sperm health	Y	N	Y	Arafa *et al.* (2020)
Impryl^®^	Goodlife pharma	Betaine 200 mg, l-cysteine 200 mg, Vit B3 16 mg, Zinc 10 mg, Vit B6 1,4 Mg, Vit B2 1.4 mg, FA 400 µg, Vit B12 2.5 µg	1/day	N	N	$49.50	Impryl^®^ is a food supplement that is recommended for men and women who want children. It is used to improve the quality of both egg cells and sperm cells.	Y	N	N	Clément *et al.* (2020)
Menevit^®^	Bayer	Vit C 100 mg,^d^ Zinc 25 mg^d^ Vit E 400 IU,^d^ FA 500 µg,^d^ Lycopene 6 mg, Garlic oil, Selenium 26 µg	1/day	Y	N	$32.99	Made with a unique combination of antioxidants, Menevit can help create a better environment, which includes protecting against DNA damage to support male fertility and sperm health.	Y	N	Y	Tremellen *et al.* (2007, 2021) and Tunc *et al.* (2009) Price from lifepharmacy.co.nz
Profertil^®^	Lenus pharma	l-Carnitine 440 mg, l-arginine 250 mg, Vit E 120 mg,^d^ Glutathione 80 mg, Selenium 60 µg,^d^ Coenzyme Q10 15 mg, FA 800 µg^d^	2/day	Y	N	$69.00	Significantly improves all parameters in sperm necessary for successful conception and pregnancy: motility, morphology, count, and volume of sperm; significant reduction of DNA fragmentation in sperm cells. Pregnancy rate: avg. 26–41%	Y	N	Y (partly: pregnancy rate of 41% not reported in studies)	Lipovac *et al.* (2016, 2021), Schauer *et al.*, Lipovac (2014), and Imhof *et al.* (2012)
Proxeed men^®^	Sigma Tau	l-Carnitine 1770 mg, Acetyl-l-carnitine 500 mg, Propionyl-l-carnitine 252 mg, l-arginine 1 g, Vit C 90 mg,^d^ Coenzyme Q10 20 mg, Vit E 12 mg,^d^ Selenium 50 µg,^d^ Zinc 7.5 mg,^d^ FA 200 µg, Vit D 3.75 µg, Vit B12 1.5 µg^d^	1–2/day	Y (if 2/day)	N	NR	Enhancing sperm energy metabolism; increasing sperm quality: sperm count, motility, speed, and morphology; protecting sperm from oxidative stress; designed to support healthy sperm development and maximize a man’s chances of fathering a child.	Y	N	Y (partly: only improvement in sperm progressive motility in included study)	Busetto *et al.* (2012)
Proxeed Plus^®^	Sigma Tau	l-Carnitine 1 g, Acetyl-l-carnitine 500 mg, Vit C 90 mg, Zinc 10 mg, FA 200 µg, Selenium 50 µg, Coenzyme Q10 20 mg, Vit B12 1.5 µg	2/day	N	N	$ 100.00	Enhancing sperm energy metabolism; Increasing sperm quality: sperm count, motility, speed, and morphology; protecting sperm from oxidative stress; designed to support healthy sperm development and maximize a man’s chances of fathering a child.	Y	Y	Y (definition of ‘sperm speed’ not clear)	Szymański *et al.* (2021), Micic *et al.* (2019), and Busetto *et al.* (2018)
Tetrafolic^®^	Nurilia	N-acetyl l-Cysteine 50 mg, Betaine 40 mg, Methionine 40 mg, l-cystine 35 mg, l-glutamine 35 mg, l-glycine 35 mg, Vit B3 16 mg, Zinc 14 mg,^d^ Vit B2 1.4 mg,^d^ Vit B6 1.4 mg,^d^ FA 500 µg,^d^ Vit B12 2.5 µg^d^	1/day	Y	N	$31.00	Many couples experience fertility problems or experience repeated miscarriages. Fighting the effects of oxidative stress and blocking the effects of the MTHFR mutation (often the cause of fertility problems) can help restore optimal fertility.	Y	N	NA (claim not directly related to the product)	Clément *et al.* (2020)
Vitascience speramax^®^	Vitascience USA	Vit E 14 IU, FA 400 µg, Zinc 8 mg, Selenium 40 µg, l-carnitine 500 mg	1/day	N	N	$35.00	A blend of vitamins for male fertility to support sperm motility, strength, and volume	N	Y	N	Hamzah (2018)
Gametix M^®^	Densmore laboratoire	Carnitine 2 g, Taurine 250 mg, Coenzyme Q10 20 mg, Zinc 10 mg, Selenium 50 µg, Vit C 180 mg,^d^ Vit B3 16 mg, Vit E 12 mg, Vit B6 1.4 mg,^d^ FA 200 µg	1/day	Y	N	$26.19	Contributes to normal fertility, reproduction, and spermatogenesis.	N	N	N	NA
FertilHom^®^	Natura-medicatrix	l-Carnitine 2.8 g, Acetyl-l-carnitine 500 mg, l-arginine 250 mg, Glutathione 100 mg, Coenzyme Q10 40 mg, Zinc 7.5 mg, FA 200 µg, Selenium 50 µg, Vit B12 2 µg	1/day	N	N	$ 74.85	FertilHom makes it possible to increase the number of spermatozoa and their mobility.	N	N	N	NA
Ready.Set.Go!^®^	Best nest wellness	Vit A 1200 µg,^d^ Vit C 60 mg, Vit D 10 µg, Vit E 34 mg,^d^ Vit K 80 µg, Vit B1 1.5 mg,^d^ Vit B2 1.5 mg,^d^ Vit B3 20 mg,^d^ Vit B6 2 mg,^d^ FA 400 µg, Vit B12 6 µg,^d^ Vit B5 10 mg,^d^ magnesium 50 mg, Zinc 30 mg,^d^ Selenium 100 µg,^d^ Copper 2 g,^d^ Manganese 2 mg, Chromium 120 µg^d^	1/day	Y	N	$ 35.00	Helps Nourish you and supports fertility goals	N	N	N	NA
Belimen^®^	Beli	Vit C 100 mg,^d^ Vit D3 50 µg,^d^ Vit E 134 mg,^d^ Vit B6 2 mg,^d^ FA 1020 µg^e^, Vit B12 14 µg,^d^ Zinc 15 mg,^d^ Selenium 70 µg,^d^l-carnitine 100 mg, l-arginine 200 mg, Coenzyme Q10 100 mg, l-taurine 110 mg, N-acetyl-l-cysteine 25 mg	2/day	Y	Y	$ 50.00	Increase sperm count; Protect sperm while they grow; Improve sperm quality; Improve egg penetration; Balance acid-alkaline in the body to help sperm survive and work properly; Boost energy; Improve cellular health	N	N	N	NA
Men’s fertility support^®^	Blue Stork	Vit B3 16 mg, Vit B6 5 mg,^d^ FA 400 µg, Vit B12 20 µg,^d^ Zinc 22 mg,^d^ Acetyl-l-carnitine NR, N-acetyl-l-cysteine NR	2/day	Y	N	$40.00	Supports healthy sperm	N	N	N	NA
Legacy male fertility supplement^®^	Give legacy	Coenzyme Q10 300 mg, Vit C 250 mg,^d^ Vit D 35 µg,^d^ Vit E 100 mg,^d^ FA 1 mg,^d^ Zinc 30 mg,^d^ Selenium 70 µg,^d^l-carnitine 200 mg, D-aspartic acid 50 mg, Lycopene 5 mg	2/day	Y	N	$65.00	A science-backed multivitamin developed by experts to support healthy sperm production and fertility.	N	N	N	NA
Natalist prenatal for him^®^	Natalist	Vit C 500 mg,^d^ Vit D 10 µg, Vit E 268 mg,^d^ Coenzyme Q10 200 mg, Lycopene 8 mg, DHA 450 mg	4/day	Y	N	$55.00	When combined with a healthy diet and lifestyle, the ingredients in our extensively researched and scientifically formulated Prenatal for him have been shown to improve semen volume and sperm quality.	N	N	N	NA
Theralogix ConceptionXR Reproductive Health Formula/Motility Support Formula^®^	Theralogix	Vit C 500 mg,^d^ Vit D 25 µg,^d^ Vit E 67 mg,^d^ FA 1000 µg,^d^ Zinc 20 mg,^d^ Selenium 200 µg,^d^ Lycopene 10 mg	2/day	Y	N	$25.33	ConceptionXR Reproductive Health Formula is designed to support sperm health and help you do your part to promote a healthy pregnancy.	N	N	N	NA
EU natural conception men^®^	Eu natural	Vit C 60 mg, Vit D 4000 IU,^d^ Vit E 60 IU,^d^ Vit B1 15 mg,^d^ Vit B2 17 mg,^d^ Vit B3 10 mg, Vit B6 10 mg,^d^ FA 800 µg,^d^ Vit B12 120 µg,^d^ Vit B5 10 mg,^d^ Iodine 150 µg, Zinc 30 mg,^d^ Selenium 140 µg,^d^ Manganese 2 mg, Chromium 120 µg^d^	2/day	Y	N	$29.99	Our science-backed formula promotes healthy sperm motility to support a conception.	N	N	N	NA
One a day pre pregnancy men^®^	One a day	Vit A 810 µg, Vit C 100 mg,^d^ Vit D 20 µg,^d^ Vit E 45 mg,^d^ Vit B1 1.2 mg, Vit B2 1.3 mg, Vit B3 16 mg, Vit B6 1.3 mg, FA 500 µg,^d^ Vit B12 2.4 µg, Biotin 30 µg, Vit B5 5 mg, Calcium 260 mg, Iodine 150 µg, Magnesium 140 mg, Zinc 15 mg,^d^ Selenium 55 µg, Copper 0.9 mg, Manganese 2.3 mg, Chromium 35 µg, Lycopene 6 mg	1/day	Y	N	NR (only price for combination with female supplement)	For future dads, a complete multivitamin specially formulated with powerful antioxidants to support healthy sperm.	N	N	N	NA
Baby ASAP for men^®^	Emerald Bay labs	Vit C 200 mg,^d^ Vit E 400 IU,^d^ Vit B6 100 mg,^d^ FA 800 µg,^d^ Vit B12 1000 µg,^d^ Magnesium 50 mg, Zinc 50 mg,^d^ Selenium 200 µg,^d^ Coenzyme Q10 200 mg, Diindolylmethane 50 mg, R-Alpha lipoic acid 50 mg,^d^ Lycopene 4 mg	4/day	Y	N	NR	Premium male fertility supplement. Support for low sperm count, low sperm motility, and sperm morphology, for sperm count and quality, improved hormone balance	N	N	N	NA
FertilPro men^®^	Yad tech	l-Carnitine 400 mg, Vit C 300 mg,^d^ Vit E 67 mg,^d^ FA 1 mg,^d^ Vit B12 50 µg,^d^ Zinc 30 mg,^d^ Selenium 80 µg^d^	1/day	Y	N	$26.66	This supplement combines the power of l-carnitine with vitamins A, C, E, B12 and minerals selenium and zinc to help men maintain optimum health.	N	N	N	NA
Evolution 60^®^	Pregnitude/Everett laboratories	Vit E 27 IU, FA 200 µg, Selenium 55 µg, Myoinositol 1000 mg, l-carnitine 30 mg, l-arginine 30 mg	1/day	Y	N	NR	Evolution60 supplies nutrients to help promote a healthy male reproductive system.	N	N	N	NA
NHP Advanced fertility support for men^®^	Natural health practice	l-Arginine 300 mg, Vit E 161 mg,^d^ Vit C 200 mg,^d^ Zinc 30 mg,^d^ Magnesium 20 mg, l-carnitine 100 mg, l-taurine 100 mg, Coenzyme Q10 60 mg, Calcium 20 mg, Beta carotene 5 mg, Vit B1 20 mg,^d^ Iron 5 mg, Manganese 5 mg,^d^ Vit B6 20 mg,^d^ Vit B5 20 mg,^d^ Vit B2 20 mg,^d^ Vit B3 20 mg,^d^ Selenium 100 µg,^d^ Vit A 696 µg, Vit D3 10 µg, Chromium 20 µg, FA 400 µg, Vit B12 20 µg^d^	3/day	Y	N	$41.34	Highest possible quality to ensure maximum effectiveness in the quickest amount of time. Greatly helps to increase chances of conception both naturally and by IVF.	N	N	N	NA
Androenergen^®^	Ovaterra (Fertility nutraceuticals)	Vit E 20 µg, Coenzyme Q10 300 mg	3/day	N	N	$159.64	AndroEnergen™ Ubiquinol CoQ10 is a nutritional supplement of clinical-grade coenzyme Q10 in the active ubiquinol form to help maintain male reproductive health by supporting sperm health.	N	N	N	NA
Fertilmas^®^	Supplemena	l-Carnitine 408 mg, l-arginine 400 mg, Vit C 120 mg,^d^ Vit E 13 mg, Zinc 15 mg,^d^ Coenzyme Q10 15 mg, Vit B6 2.2 mg,^d^ Vit D 4 µg, FA 600 µg,^d^ Selenium 56 µg,^d^ Vit B12 3.4 µg^d^	2/day	Y	N	$79.00	Fertilmas, our premium male fertility supplement, was formulated to support sperm maturation in men with sub-optimal sperm.	N	N	N	NA
Andromas^®^	Effik	Myoinositol 4 g, Coenzyme Q10 200 mg, Zinc 20 mg,^d^ Vit D3 25 µg,^d^ FA 400 µg, Selenium 105 µg^d^	1/day	Y	N	$ 73.19	Contributes to spermatogenesis, normal fertility and reproduction.^c^	N	N	N	NA
Gestagyn Men^®^	Gynea/KernPharma	Astaxanthin 8 mg, DHA 500 mg, Zinc 10 mg, Selenium 55 µg, Vit E 12 mg, Melatonin 0.5 mg, Coenzyme Q10 100 mg, FA 200 µg, Vit B12 2.5 µg	1/day	N	N	$31.45	Food supplement specially formulated for male fertility.	N	N	N	NA
Seidiferty^®^	Lab Seid	DHA 1000 mg, Coenzyme Q10 200 mg, Zinc 22.5 mg,^d^ Selenium 105 µg^d^	2/day	Y	N	$83.90	Food supplement that improves sperm morphology, concentration, and motility in infertile men. It decreases oxidative stress, maintains the integrity of the cell membrane and reduces DNA fragmentation.	N	N	N	NA
Vitafertil^®^	Sana expert	Vit C 100 mg,^d^ Vit B1 2 mg,^d^ Vit B12 4 µg,^d^ Vit B6 3 mg,^d^ FA 200 µg, Magnesium 100 mg, Zinc 15 mg,^d^ Selenium 126 µg,^d^ Copper 1 mg,^d^l-arginine 800 mg, Alpha lipoic acid 6 mg,^d^ Coenzyme Q10 10 mg	2/day	Y	N	$21.76	SanaExpert VitaFertil is a dietary supplement that contains important micronutrients to support male reproduction and fertility.	N	N	N	NA

a Direct citations from the product website.

b Improved male fertility or comparable statements are defined by the review authors as a significantly higher clinical pregnancy rate or live birth rate after intervention. For readability the level of evidence, i.e. the quality of the studies, has not been added to this table, as it can be found in more detail in Table 2.

c Translated to English from Spanish.

d Substance exceeding RDA.

e Substance exceeding UL.

DHA, docosahexaenoic acid; FA, folic acid; NA, not applicable; NR, not reported; RDA, recommended dietary allowance; UL, tolerable upper intake level; Vit, vitamin.

The general health claim of all manufacturers was that their product ‘optimizes’, ‘normalizes’, ‘improves’, or ‘supports’ male fertility or reproduction. Both groups of supplements, with and without published studies, made these claims. Three manufacturers with a supplement tested in a clinical trial mentioned the study in their claims with terms as ‘shown in a clinical study’, ‘tested effective’, and ‘significantly improves’. Three manufacturers selling a supplement without published evidence referred to science in their claims with terms such as ‘a science-backed multivitamin’, ‘scientifically formulated’, and ‘science-backed formula’.

### Included studies

We found published studies for 13 of the 34 supplements, while for one supplement (Speramax^®^), we found only an animal study testing the supplement in female mice (Hamzah, 2018). Most study designs were pre–post studies with or without a (retrospective) control group treated with placebo or receiving no treatment. We also identified five RCTs, of which two investigated the supplement Proxeed Plus^®^ (Tremellen *et al.*, 2007; Clifton and Ellington 2009; Busetto *et al.*, 2018; Tsounapi *et al.*, 2018; Micic *et al.*, 2019). In addition, we found one case report, one retrospective cohort study and one non-randomized clinical study (Junca *et al.*, 2012; Lipovac *et al.*, 2016; Tremellen *et al.*, 2021). Most studies included male fertility patients, based on abnormal semen parameters or high DNA fragmentation. The number of randomized or included patients ranged from one couple to 657 participants. The treatment period ranged from 30 days to 6 months (Table 2).

**Table 2.** Characteristics of included studies.

Supplement name	Author, year	Study type	Type of participants	No. of participants	Treatment period	Outcome parameters	Results	Risk of bias	RoB tool
Androferti^®^	Nazari *et al.*, (2021)	Pre–post without control group	Idiopathic oligoasthenoteratozoospermia	70	3 months	Semen parameters	Significant improvement of sperm concentration and morphology	Fair study quality	NIH
	Abad *et al.*, 2013	Pre–post without control group	Asthenoteratozoospermia	20	3 months	Semen parameters, sDF (SCD), biochemical and ongoing pregnancy	Significant improvement of sperm A and A + B motility, vitality and morphology	Fair study quality	NIH
	Goodwin *et al.*, 2019	Pre–post without control group	Abnormal semen parameters	120	Minimum 30 days	Semen parameters	Significant improvement of sperm total and progressive motility	Poor study quality	NIH
	Balmori Boticario *et al.*, 2010	Pre–post without control group	Repeated ART failure, oligozoospermia or asthenozoopsermia, and sDF > 20%	101	70 days	Semen parameters, sDF (TUNEL)	Significant improvement of sDF	Fair study quality	NIH
	Ballescá *et al.*, 2012	Pre–post without control group	Idiopathic asthenoteratozoospermia	69	3 months	Semen parameters, clinical (?) pregnancy, adverse events	Significant improvement of sperm A and A + B motility	Poor study quality	NIH
	López Granollers *et al.*, 2011	Pre–post without control group	Not described	65	Minimum 30 days	sDF (SCD), sperm nuclear vacuoles	Significant improvement of sDF	Poor study quality	NIH
Condensyl^®^	Dattilo *et al.*, 2014	Pre–post without control group	At least 2 ART failures and sDF >20%	84	4 months	Semen parameters, sDF (TUNEL), SDI, clinical pregnancy, LBR	Significant improvement of sDF and SDI	Poor study quality	NIH
	Junca *et al.*, 2012	Case report	Couple with multiple ART failures	1 couple	6 months	SHBA, sDF (TUNEL), SDI, clinical pregnancy, LBR	Improvement of sDF and SDI	na	na
Condensyl, Fertibiol^®^	Amar *et al.*, 2015	Pre–post with control group	Infertile men (>3 years) with ART failure and high sDF (no cut-off reported)	304	5 weeks Fertibiol, 4 months Condensyl	Semen parameters, sDF (TUNEL), SDI, clinical pregnancy, miscarriage, LBR	Significant improvement of sDF SDI, clinical pregnancy rate and live birth rate	High risk of bias	EPOC
Cyclofert^®^	Elmahaishi *et al.*, 2017	Pre–post with control group	Infertile men with at least one abnormal semen parameter	600	6 months	Semen parameters	Significant improvement of sperm concentration, motility and morphology	High risk of bias	EPOC
Fertilaid for men^®^	Clifton and Ellington 2009	RCT	Men with abnormal semen parameters	20	90 days	Semen parameters	Significant improvement of normal motile sperm number	High risk of bias	Cochrane RoB
Fertilovit^®^	Wirleitner *et al.*, 2012	Pre–post without control group	Men undergoing IMSI	147	2–12 months	Semen parameters	Significantly lower sperm volume and grade D motility, significant improvement of TMSC	Fair study quality	NIH
Fertimax2^®^	Kacem *et al.*, 2014	Pre–post without control group	Men undergoing IVF or ICSI treatment	48	2–5 months	Semen parameters, embryological parameters, biochemical and clinical pregnancy, LBR	Significant improvement of fertility rate, cleavage rate and embryo quality	Poor study quality	NIH
FH Pro for men^®^	Arafa *et al.*, 2020	Pre–post without control group	Men with idiopathic and unexplained infertility	150	3 months	Semen parameters, sDF (SCD), ORP, adverse events	Significant improvement of sperm progressive motility, sDF, and ORP in both patient groups	Fair study quality	NIH
Menevit^®^	Tunc *et al.*, 2009	Pre–post without control group	Men with sperm oxidative stress (assessed with NBT assay)	50	3 months	Semen parameters, serum hormones, sDF (TUNEL), sperm apoptosis and protamination semen ROS production (NBT assay)	Significant improvement of sDF	Fair study quality	NIH
	Tremellen *et al.*, 2021	Retrospective cohort	Couples undergoing first IVF/ICSI cycle	657	Unknown	Semen parameters, embryo quality, biochemical and clinical pregnancy, LBR	Significantly more clinical pregnancies and live births in Menevit group	Poor study quality	NOS
	Tremellen *et al.*, 2007	RCT	Couples undergoing IVF/ICSI, sperm oxidative stress, and sDF >25%	60	3 months	IVF outcomes (e.g. number and quality of embryos), biochemical and clinical pregnancy, miscarriage	Significantly higher clinical pregnancy rate and implantation rate	Low risk of bias	Cochrane RoB
Profertil^®^	Imhof *et al.*, 2012	Pre–post with control group	Infertile men with at least one abnormal semen parameter	214	3 months	Semen parameters, clinical pregnancy	Significant improvement of sperm volume, concentration, motility and morphology	High risk of bias	EPOC
	Lipovac, 2014	Pre–post with control group	Sub-/infertile men with abnormal semen analysis	116	3 months	SHBA	Significant improvement of SHBA	High risk of bias	EPOC
	Lipovac *et al.*, 2021	Pre–post with control group	Infertile men with abnormal semen analysis	339	3 months	sDF (SCD), pregnancy (unclear)	Significant improvement of sDF	High risk of bias	EPOC
	Lipovac *et al.*, 2016	Non-randomized clinical trial	Subfertile men with at least one pathologic semen parameter	299	3 months	Semen parameters	Significant improvement of semen parameters in Profertil and l-carnitine monotherapy group	Critical risk of bias	ROBINS II
	Tsounapi *et al.*, 2018	RCT	Infertile men with idiopathic oligoasthenozoospemia	217	90 days	Semen parameters, serum hormones, sDF (SCSA), sperm hyperactivation, hypoosmotic swelling test, seminal zinc, clinical pregnancy	Significant improvement of sperm motility	High risk of bias	Cochrane RoB
Proxeed Plus^®^	Szymański *et al.*, 2021	Pre–post without control group	Idiopathic male fertility patients	78	6 months	Semen parameters	Significant improvement of sperm concentration and motility	Poor study quality	NIH
	Micic *et al.*, 2019	RCT	Infertile men with idiopathic oligoasthenozoospermia	175	6 months	Semen parameters, sDF (SCD), α glucosidase activity, seminal l-carnitine concentration	Significant improvement of volume, motility and sDF	Low risk of bias	Cochrane RoB
	Busetto *et al.*, 2018	RCT	Infertile men at least one abnormal semen parameter and with or without varicocele	104	6 months	Semen parameters, clinical (?) pregnancy	Significant improvement of sperm concentration, motility and morphology	Moderate risk of bias	Cochrane RoB
Tetrafolic, Impryl^®^	Clément *et al.*, 2020	Pre–post without control group	MTHFR SNP C677 T carriers (men and women), tested after > 3 miscarriages, or > 3 years of infertility and 3 failed ARTs	89	3 months	Serum homocysteine	Significant decrease in serum homocysteine	Fair study quality	NIH

EPOC, effective practice and organization of care risk of bias tool; IMSI, intracytoplasmic morphologically selected sperm injection; LBR, live birth rate; na: not applicable; NBT assay, nitroblue tetrazolium assay; NIH, national institutes of health risk of bias tool; NOS, Newcastle-Ottawa scale; ORP, oxidation reduction potential; RCT, randomized controlled trial; RoB, risk of bias; ROBINS, risk of bias in non-randomized studies of interventions; ROS, reactive oxygen species; SCSA, sperm chromatin structure assay; SCD, sperm chromatin dispersion test; sDF, sperm DNA fragmentation; SDI, sperm decondensation index; SHBA, sperm hyaluronic binding assay; TMSC, total motile sperm count; TUNEL, terminal deoxynucleotidyl transferase dUTP nick end labeling assay.

### Risk of bias in studies

The overall quality of the included studies was poor (Table 2). This was due to small groups or no sample size calculation, no assessment of treatment adherence or the use of other supplements before or during the study, no (description of) blinding of outcome assessors, high or no description of loss to follow-up, and/or failure to report all predefined outcome measures. All five pre–post studies with a control group were graded as having a high risk of bias based on baseline imbalances between the study groups, the use of a retrospective control group, and/or no (report of) blinding of outcome assessors (Imhof *et al.*, 2012; Amar *et al.*, 2015; Lipovac *et al.*, 2016, 2021; Elmahaishi *et al.*, 2017). One non-randomized clinical study was graded with the ROBINS II tool as having a critical risk of bias based on no report of baseline parameters, treatment adherence or loss to follow up, and no statistical comparison of end point outcomes between the two study groups (Lipovac *et al.*, 2016). Moderate or high risk of bias was assigned to three of the five RCTs based on failure to report the method of randomization, no blinding of outcome assessors, and loss to follow-up (Clifton and Ellington 2009; Busetto *et al.*, 2018; Tsounapi *et al.*, 2018). Two RCTs were graded as having a low risk of bias with the Cochrane Risk of Bias tool (Tremellen e*t al*., 2007; Micic *et al.*, 2019).

## Discussion

This is the first scoping review on nutritional supplements that are freely available to male fertility patients. It draws an image of the broad selection subfertile men are offered when they are searching for supplements in order to improve their fertility.

There is a great variety of active substances in supplements and nearly 80% of the supplements contain substances in a dosage exceeding the recommended daily allowance. In two supplements, a substance was present in a dosage exceeding the tolerable UL. It is worth noting that both of these values for dietary reference intake are set based on heterogeneous studies in groups of healthy individuals. They should be regarded as guiding instead of binding (Institute of Medicine Subcommittee, on Interpretation, Intakes Uses of Dietary Reference, and Intakes Institute of Medicine Standing Committee on the Scientific Evaluation of Dietary Reference, 2000). Despite this fact, we chose to report these outcomes measures because of the often-overlooked risk of reductive stress.

Previous research has shown that high dosages of antioxidants can cause reductive stress (Dattilo *et al.*, 2016; Henkel *et al.*, 2019). One can imagine that if oxidative stress causes the balance to tip over, reductive stress will be able to do this the other way. When this results in very low levels of ROS, physiological processes that ensure sperm maturation and capacitation might be disturbed, leading to impaired fertility. We found no information on this risk on any of the supplement websites.

Our review furthermore shows the costs of supplements for male fertility patients. The supplements need to be taken for at least 3 months to cover the period necessary for sperm production, 72 days. This means that men pay an average of at least 159.30 US dollars, with a maximum price of 478.92 US dollars for a 3-month period.

The numbers are even more remarkable when one considers that only 38% of the supplements were tested in clinical trials which, overall, were poor in quality. Only two RCTs were found to have a low risk of bias (Tremellen *et al.*, 2007; Micic *et al.*, 2019). The recently updated Cochrane review on antioxidants for subfertile men also reports that the evidence for male fertility supplements is of low quality (de Ligny *et al.*, 2022).

Studies on the effect of a single antioxidant substance on clinical outcomes are limited, with often no more than two available studies on one substance. A low-quality study compared zinc with a placebo and found significantly more pregnancies in the intervention group (Omu *et al.*, 1998). Two >25-year-old studies compared vitamin E with placebo and one showed significantly more live births in the intervention group (low quality evidence) (Kessopoulou *et al.*, 1995; Suleiman *et al.*, 1996). l-Carnitine and co-enzyme Q10 each did not have a significant effect on clinical pregnancies or live births (Balercia *et al.*, 2005, 2009).

All these findings suggest that the subject of male fertility supplements is non-transparent, which could lead to misinformation of patients. It is important to note that (male) fertility patients are in a vulnerable situation, as the cause of their subfertility is often unclear (Fainberg and Kashanian 2019). This may lead to insecurity and the motivation to seek a solution outside conventional health care. It should be a priority to give clear information based on good quality evidence to these patients.

The discrepancy between the health claims and supporting evidence should lead to interference of regulatory institutions. In reality, the regulatory framework of dietary supplement is very complex and, in many ways, different from that of medical drugs or devices (Dwyer *et al.*, 2018). There is no clarity on the definition of dietary supplements and regulation of the market differs even between economically comparable regions such as the USA and the European Union. All these challenges aside, the aim of health care providers, but also of governments and regulatory institutions, is to ensure the safety of consumers and their ability to make an informed decision. Based on the results of this review, we can state that these goals have not yet been achieved.

Large RCTs, that check and correct for confounding factors such as lifestyle, diet, and use of other supplements are therefore urgently needed. Primary outcomes should be clinical, with the ultimate outcome being live birth rate. The previously mentioned Cochrane review on this subject concluded that antioxidants may lead to an increased live birth rate (OR 1.43, 95% confidence interval 1.07–1.91) based on 12 studies (de Ligny *et al.*, 2022). Unfortunately, the evidence for this outcome was of very low certainty. In response to this, a few well-designed studies are now being conducted, but the scientific attention still does not reflect the scale of the problem (Smits *et al.*, 2020; NCT04193358).

A limitation of this review is that supplements with ingredients listed in a language other than English, Dutch, French, Spanish, or German were excluded. This led to exclusion of several supplements (with ingredients listed in Russian and Chinese). Many supplements were excluded for containing plant extracts; this may have resulted in a less comprehensive image of available supplements. In this review, we have searched shopping websites to show a patient’s view of the market for male fertility supplements. As a consequence, a comprehensive search strategy was not possible.

## Conclusion

This review shows that the extensive range of OTC male fertility supplements is not supported by good quality evidence. To make a well-informed decision, patients should be provided with information on harms and benefits of a treatment based on well-designed RCTs, ideally with the ultimate end point that matters: ongoing pregnancy or live birth. Surveillance of the content of the supplements and the information available for patients and professionals should be improved. Factual information on supplements as provided in this review should be available for patients through professional channels such as websites of fertility clinics and patient organizations.

## Data Availability

The data underlying this article will be shared on reasonable request to the corresponding author.
